# GammaTile Brachytherapy Combined With External Beam Radiation Therapy for the Treatment of a Partially Resected Secondary Glioblastoma (WHO Grade 4 IDH-Mutant Astrocytoma): Matching External Beam Dose Gradient to Brachytherapy Dose Fall-Off

**DOI:** 10.7759/cureus.19717

**Published:** 2021-11-18

**Authors:** Matthew S Peach, Aiden M Burke, Jasmine Jo, Andrew W Ju, Kaida Yang

**Affiliations:** 1 Department of Radiation Oncology, East Carolina University Brody School of Medicine, Greenville, USA; 2 Department of Neurology, Vidant Health, Greenville, USA

**Keywords:** grade 4 astrocytoma, reirradiation, combination brachytherapy, idh-mutated, recurrent glioblastoma, radiation therapy, recurrent glioma, brachytherapy, glioblastoma, gammatile

## Abstract

Reirradiation of recurrent glioblastomas is most commonly managed with hypofractionated external beam radiation with a modest overall effect. GammaTile, which is a Cesium-131 source embedded in collagen mesh, is an approach that allows the surgical bed of resectable intracranial tumors to receive a greater biological dose than is possible with any form of external beam radiation therapy (EBRT). In this case report, a 28-year-old male presents with a WHO grade 4 isocitrate dehydrogenase (IDH)-mutant astrocytoma (formerly secondary glioblastoma) of the left occipital/parietal lobe after receiving 45 Gy and two cycles of adjuvant temozolomide four years prior for a grade 3 IDH-mutant astrocytoma. The patient proceeded to undergo craniotomy with maximal safe resection and application of GammaTile to a dose of 60 Gy at 5mm depth. Shortly afterward, he developed symptomatic progression of disease in the bilateral splenium and left thalamus/basal ganglia. We irradiated the undertreated residual disease with EBRT to a dose of 35 Gy in 10 fractions without introducing excessive dose to the GammaTile irradiated volume. This was achieved by creating one portion of the planning target volume with a homogeneous dose and another part where the delivered dose decreased with the GammaTile dose buildup. Treatment planning utilized the Gradient Optimization feathering technique with non-coplanar volumetric modulated arc therapy. The resulting composite between the hypofractionated EBRT and GammaTile dose distribution created an approximate dose equivalent of 50 Gy in 2 Gy fractions to the residual disease with no hot spots or areas of under coverage. This is the first report showing the feasibility of combining GammaTile with dose-matched EBRT volumes in a reproducible manner to sub-totally resected, recurrent intracranial neoplasms.

## Introduction

Low-grade and grade 3 gliomas recurring as WHO grade 4 isocitrate dehydrogenase (IDH)-mutant astrocytomas are rare occurrences, representing 5% of all grade 4 gliomas [1]. While the term secondary glioblastoma is no longer used per the updated WHO 2021 CNS5 classification system [2], for brevity, we will refer to grade 4 IDH-mutant astrocytoma as secondary glioblastoma (GB). When grade 3 gliomas recur as secondary GB, the disease is most often adjacent to or within the initial site [3], which in most cases has already undergone significant irradiation to doses approaching 60 Gy. As with the treatment of recurrent GB, the typical permittable doses in reirradiation scenarios for secondary transformed GB with EBRT are insufficient for durable control. GammaTile® (GT Medical Technologies, Tempe, Arizona) consists of a collagen mesh implanted with Cesium-131 seeds [4] that allows the application of a greater dose to resected tumor beds beyond what can be achieved with EBRT reirradiation. The resulting brachytherapy treatment volume is, however, limited to areas immediately adjacent to the resection cavity. In this case report, we discuss the use of GammaTile brachytherapy followed by subsequent hypofractionated EBRT to unresectable portions of a secondary GB after prior EBRT.

## Case presentation

**Figure 1:**
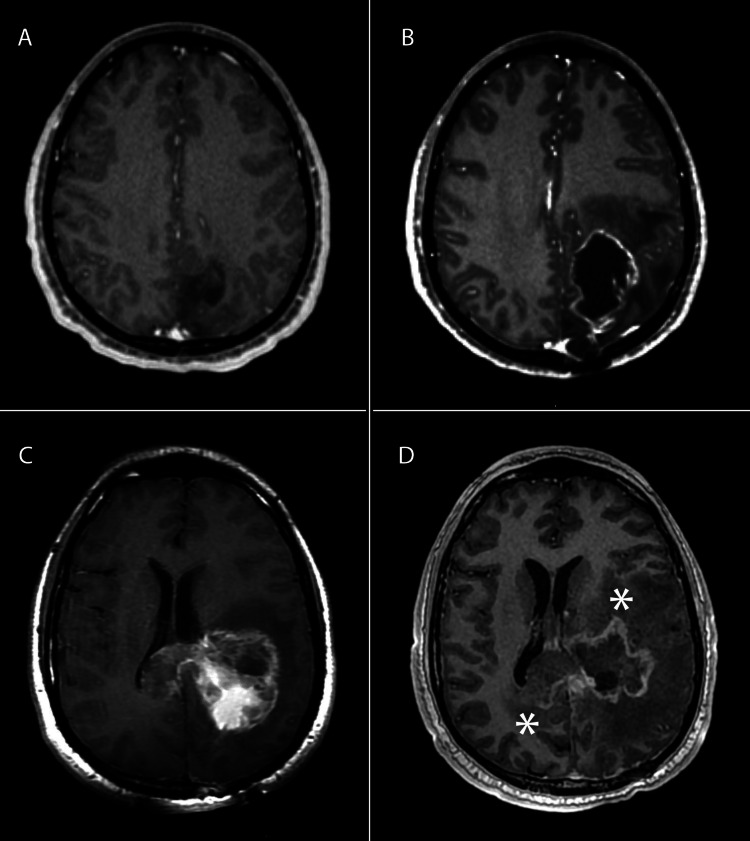
Radiographic evolution of patient’s disease. Contrast enhanced, axial T1 MRI images status post initial maximal safe resection of grade 3 astrocytoma four years prior to case presentation (A), status post second subtotal resection demonstrating GB (B), progression of secondary GB (C), status post maximal safe resection and GammaTile placement showing progression of anterior disease component and posterior internal capsule component as indicated by the asterisk (D).

The patient is a 28-year-old male with prior history of a grade 3 astrocytoma, who presented to our emergency department (ED) after having a general tonic-clonic seizure (GTC). Initially, his disease was appreciated four years prior when he presented with a GTC and was found to have a grade 3 astrocytoma of the left occipital lobe (Figure 1A). He underwent maximal safe resection followed by adjuvant EBRT to 45 Gy in 25 fractions at an outside institution. Due to reasons outside the patient’s control, he received only two cycles of adjuvant temozolomide following EBRT. He developed disease recurrence, presenting as a GTC six months prior to this ED presentation, where imaging demonstrated tumor progression with increased extension into the parietal lobe (Figure 1B). He subsequently underwent a second maximal safe resection later that month. Final pathology returned as GB. Follow-up imaging four months afterward demonstrated disease progression and he was started on bevacizumab as well as a tumor treatment field (TTF) device. He unfortunately only tolerated TTFs for one month, ending one month prior to the ED presentation.

Upon admission to our hospital from the ED, MRI demonstrated progression of disease in the left occipital-parietal lobes with extension into the splenium and anterior-inferior extension into the left thalamus and basal ganglia (Figure 1C). His physical exam was notable for mild right-hand weakness, but he was otherwise neurologically intact. Despite changes to his antiepileptic medication, he had seizure recurrence a few weeks following admission. Presuming that the area of tumor recurrence received a definitive dose in the past, the consensus decision was to proceed with repeat maximal safe resection with GammaTile placement. A dose of 60 Gy was prescribed to a 5 mm depth using a total of eight tiles, each containing four Cesium-131 3.5U seeds, to line the post-operative cavity volume of 17.6 cc. A significant portion of the occipital-parietal disease was debulked, with final pathology again demonstrating a grade 4 astrocytoma with molecular studies indicating an IDH-mutated, ATRX mutated, and MGMT promoter methylated phenotype with hypermutation. Postoperatively he was noted to have right upper and lower extremity weakness/spasticity with right foot drop and mild right face weakness. He required a cane to assist with ambulation and reported word-finding difficulties and decreased short-term memory. He was planning to start adjuvant temozolomide; however, he developed a severe GTC with increased muscle weakness and altered mental status two months later. MRI following this episode demonstrated mildly increased enhancement to the tissue surrounding the surgical bed with the progression of disease in the splenium and left thalamus/basal ganglia (Figure 1D).

**Figure 2:**
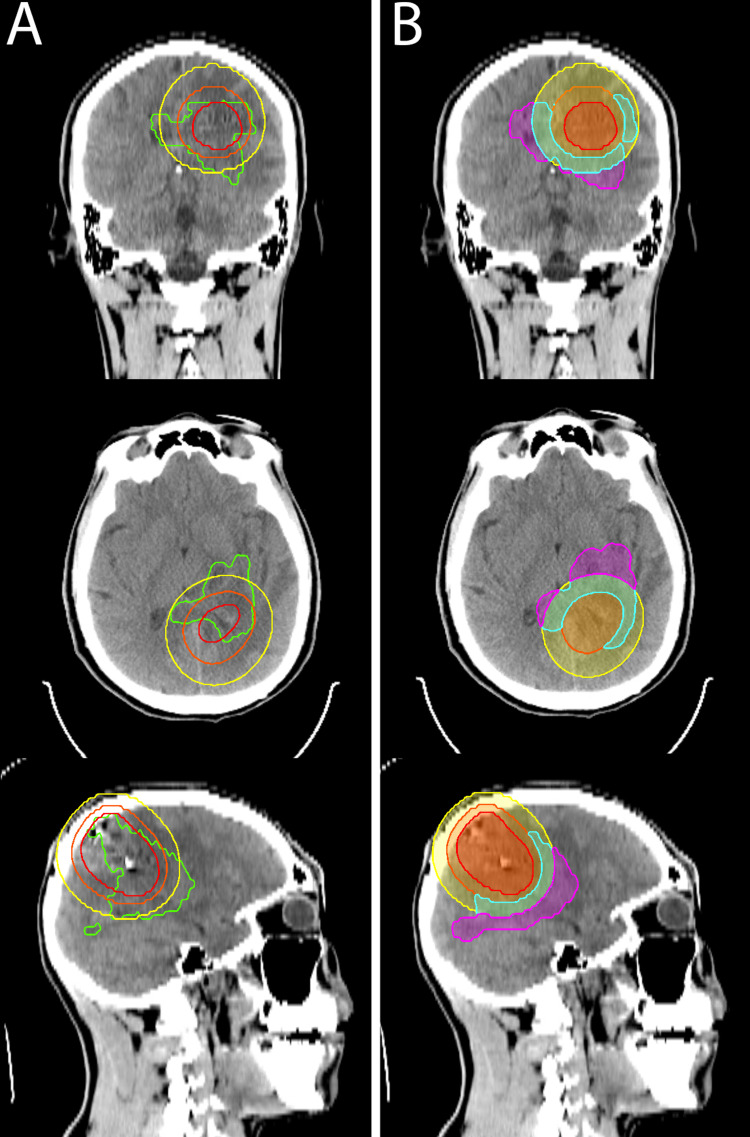
Development of post GammaTile contours and planning target volumes. Figure 2A (Left) shows the GTV (green line) on coronal, axial and sagittal CT images against the 17.5 Gy (yellow line), 35 Gy (orange line) and 60 Gy (red line) isodose lines from GammaTile delivery. Figure 2B (right) shows the ultimate planning volume consisting of two PTVs. PTV-1 (shaded magenta) overlaps with tissue that received less than 17.5 Gy from GammaTile (no shade) and PTV-2 (light blue shade) overlaps with tissue that received 17.5 Gy to up to 35 Gy (shaded yellow). The volume that received 35 Gy or more (shaded orange and red) were not included in the PTVs. Volumes and contours displayed in Velocity™ (Varian Medical Systems, Palo Alto, California).

**Figure 3:**
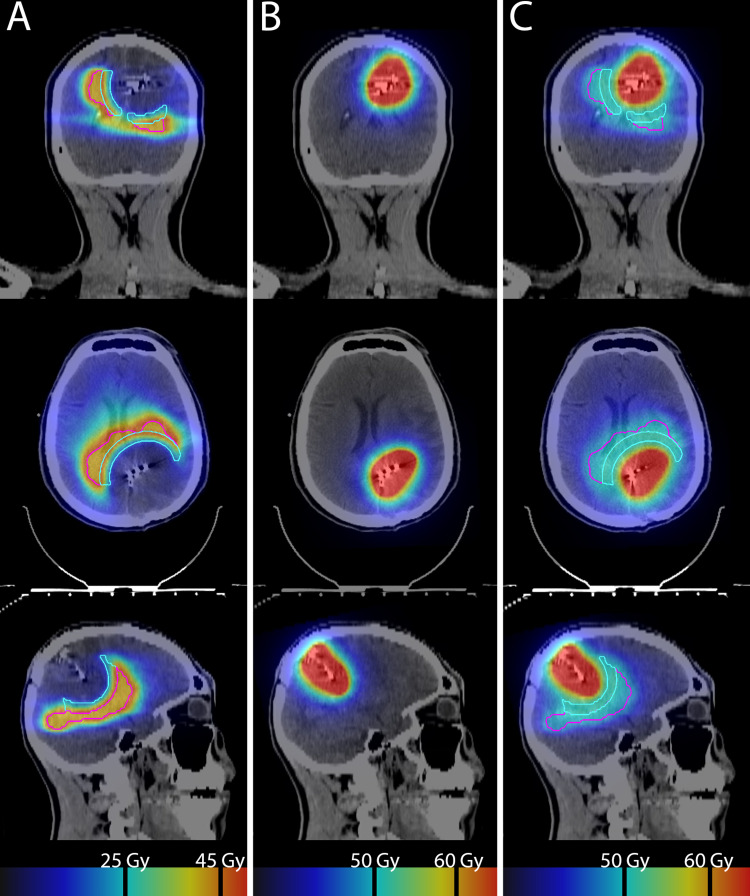
Separate and composite plans of the GammaTile dose supplemented by EBRT. Figure 3A (left) shows an IMRT arc plan with a homogenous dose in PTV1 (red line) with an EQD2 of around 45 Gy and a gradual fade to negligible dose inward from 45 Gy EQD2 in PTV2 (teal line) on coronal, axial, and sagittal CT images. Figure 3B (middle) displays moderate dose fall-off from the 60 Gy prescription isodose line of the GammaTile plan. Figure 3C (right) shows the composite between GammaTile and the EBRT volume, with a homogenous dose achieved to both PTV1 (red line) and PTV2 (teal line) around an EQD2 of 50 Gy with no hot spots or underdosing present. Display of both individual and composite plans and EQD2 conversion were accomplished in Velocity™ (Varian Medical Systems, Palo Alto, California).

Given this symptomatic disease progression, a treatment plan was made to take the progressive regions of disease outside the irradiated GammaTile volume to 35 Gy in 10 fractions. As shown in Figure 2, an initial planning target volume (PTV) was delineated consisting of the T1 post-contrast-enhancing disease with a 5 mm margin. The volume that received greater than 35 Gy from the GammaTile treatment was excluded from the PTV. The PTV was then separated into two portions by subdividing the remaining volume between tissue that received less than 17.5 Gy (PTV1), and that which received 17.5-35 Gy (PTV2). Using a volumetric modulated arc therapy plan with five arcs, one non-co-planar, a homogeneous dose of 35 Gy in 10 fractions was delivered to PTV1. This dose was calculated to have a biologically equivalent dose in 2 Gy fractions (EQD2) of 45 Gy. Dose painting gradually decreased the dose from 35 Gy to as low a dose as achievable approaching the resection bed border of PTV2 (Figure 3A). A composite of the GammaTile dose volume (Figure 3B) with a dose delivered from PTV1 and PTV2 resulted in a homogeneous dose of approximately 50 Gy EQD2 to the residual disease extending into the splenium and the left thalamus/basal ganglia (Figure 3C). Digital imaging and communications in medicine (DICOM)-RT data from the initial 45 Gy delivered from the outside institution were obtained, and cumulative dose to critical organs at risk (OARs), including the brainstem, ocular structures, and cochlea, were within established constraints.

Due to the hypermutation phenotype of his tumor, the patient was started on CCNU (100 mg/m2) and completed his EBRT with no issue or progression of his current neurological symptoms. He was last seen in follow-up by our team three months after his most recent EBRT, at which point he reported significant improvement in the right leg tremors/spasms and improved headache. He demonstrated decreased right foot drop but continued to have word-finding difficulties and short-term memory deficits. He has not developed any new areas of muscle weakness or paresthesia, visual loss, or other forms of altered sensoria. The current plan is to complete six cycles of CCNU with alternative systemic therapy thereafter upon disease progression. While temozolomide could be used to this effect, the high mutational burden found in his most recently resected disease would permit entry into currently available trials of immunotherapy.

## Discussion

The risk of radionecrosis increases with the increasing volume of brain parenchyma receiving greater than 60 Gy [5], with an even greater risk of morbidity with lower doses to subsites such as the brainstem and optic structures. The majority of EBRT regimens used to treat recurrent GB generally use a lower radiation dose than the initial course of therapy. Currently, the most common fractionation schema for reirradiation is hypofractionated, delivering between 30 Gy to 45 Gy [6]. The observed effect of these hypofractionated regimens is to improve progression-free survival (PFS) without improvement in overall survival (OS). RTOG 1205 was a Phase II trial that randomized patients with recurrent glioblastoma to bevacizumab with or without re-irradiation to 35 Gy in 10 fractions [7]. Patients in both arms had a similar median survival time of approximately 10 months, but those who underwent the re-irradiation did have improved PFS. Gamma Knife (Elekta) and LINAC-based stereotactic radiosurgery (SRS) have also been used for reirradiation of select GB patients. When compared to standard and hypofractionated EBRT regimens, SRS appears to result in improved OS, with several retrospective single-institution reports demonstrating median OS reaching 14.5 to 30 months [8]. A major drawback is that SRS can only be used for small lesions, with a significant increase in radionecrosis for larger volumes.

In contrast to the aforementioned techniques, surgery combined with brachytherapy in the form of GammaTile allows dose escalation to the tumor bed that is not achievable with EBRT or SRS. GammaTile brachytherapy also permits for substantially more sparing of critical intracranial OARs since there is no entry dose, and dose fall-off from the source is rapid. Since FDA approval in July of 2018 GammaTile has already been successfully applied in the reirradiation of a near-totally resected recurrent GB [9]. In this particular report, the recurrent lesion was localized with functional mapping and was of a size and simple geometry that permitted areas adjacent to the resected tumor to receive the adequate dose. In our present case report, the recurrent disease was irregularly shaped, with extension into eloquent areas of the brain that precluded resection. Post-implant dosimetry indicated that these areas of extension did not receive a significant dose, resulting in continued disease progression. The dose-escalation combined with gross tumor resection offered by GammaTile may result in improved OS in recurrent GB similar to what appears to result with SRS. However, any gains made for this patient from GammaTile would likely be offset by poor control of the remaining disease. Given that secondary IDH-mutated GB [10] and GB in younger patients [11] often have a more prolonged disease course, control of his disease extending from this resection bed would also likely have a significant impact on quality of life. Therefore, the decision was made to treat the underdosed areas of disease. We acknowledge that we will need further patient observation and additional cases in order to determine if multimodal brachytherapy and EBRT combined with subtotal resection confer an overall progression-free survival in recurrent glioma cases. With additional cases, our goal is to present a future case series to this end.

We opted to utilize 35 Gy in 10 fractions as our EBRT regimen, with 5 mm margins on enhancing disease [7]. We also wanted to match the EQD2 of the fractionation scheme with the dose fall-off from the GammaTile implant so that there were no hot spots, given the increased risk of radionecrosis seen with higher doses in this setting [12]. With a short timeframe allotted to plan the EBRT volume in light of the patient’s condition, two assumptions were made in regard to creating a plan that matched the dose fall-off. First, we assumed that the dose distribution from the Cesium-131 sources did not shift or drift over time. Overlaying the simulation CT with the post-GammaTile CT in this particular case demonstrated a minimal change in the Cesium seed location or resection cavity volume. Given that the majority of the Cesium-131 dose is delivered in the first two weeks post-op, we chose to plan off the original dose distribution from the post-procedure imaging. This would also maximize safety, as any contraction of the resection cavity would result in a smaller volume covered by the Cesium dose cloud. Planning off the post-procedure CT would therefore assume that the peripheral tissue had received the maximal dose from GammaTile, and the highest possible composite dose with overlapping EBRT.

The second assumption we made was that we were going to accept the GammaTile dose delivered as equivalent to EBRT in EQD2. American Association of Physicists in Medicine (AAPM) Task Group 137 guided for the radiobiological framework to convert the dose from implanted sources into external beam equivalents for prostate permanent implant brachytherapy [13]. Using the BED/EQD2 radiobiological model in this Task Group we were able to calculate that the prescription dose (60 Gy) has a biological effect of approximately 60 Gy in 2 Gy fractions. We also calculated that when GammaTile doses were less than 60 Gy, the ratio of GammaTile dose and its radiobiological effect in EQD2 was less than 1:1, i.e., the EQD2 dose was slightly less than GammaTile equivalent. The software we used for image registration and composite plan analysis included Velocity™ v4.1 (Varian Medical Systems, Palo Alto, California) and MIM MAESTRO v6.8.1 (MIM Software, Cleveland, Ohio). The image registration software was validated for rigid registration per Task Group 132 [14]. Software limitations and time constraints related to the patient’s worsening condition prevented us from converting the GammaTile dose cloud into the true EQD2. With these restrictions, the safest way to match EBRT volumes without underestimation the composite dose was to assume a 1:1 GammaTile to EQD2 dose biologic effect. We acknowledge that the radiobiological model recommended in TG-137 leads to variability based on the model parameters, and that there is significant radiobiological debate when converting permanent radioactive sources to EBRT equivalence [15-17]. This discussion is beyond the scope of this case report and will be elaborated on in a future publication.

To match the EBRT volume to the GammaTile dose fall-off such that there were no hot spots or underdosing, we utilized a method familiar to our clinic for feathering matching IMRT beams for creating craniospinal irradiation plans. Specifically, we used the Gradient Optimization feathering technique demonstrated by Maddalo et al. [18]. Our goal was to keep the composite dose to the areas of residual disease to 45-50 Gy EQD2 to avoid radionecrosis. As described above, the volume receiving 35 Gy or more dose from the GammaTile implantation was removed from a preliminary PTV. The remaining PTV was subdivided by the GammaTile isodose line that was ½ the EBRT prescription dose, in this case, 17.5 Gy, with the inner PTV feathered. Treatment planning using the aforementioned technique created a homogeneous dose to the peripheral PTV1, while a gradient from 35 Gy to as low a dose as possibly achievable for the inner volume PTV2. The EQD2 composite as displayed in Velocity™ (Varian Medical Systems, Palo Alto, California) for the GammaTile dose volume with the EBRT plan demonstrates the negligible contribution of the EBRT dose to the regions of the resection bed receiving moderate and high dose from GammaTile. Conversely, the low-dose GammaTile spill contributed a little to the overlapping EBRT volume, with a homogenous dose with an EQD2 around 50 Gy being the final result (Figure 3C). This reproducible strategy of volume generation and planning successfully permitted the conformal coverage of disease extension that had complex geometry, while not creating hot spots in or around the previously irradiated moderate to high-dose GammaTile volume.

## Conclusions

We have reported on what is the first published case of matching EBRT to GammaTile for the purpose or treating a recurrent intracranial malignancy with areas of unresectable disease that would be underdosed by the GammaTile component. This is also the first report of applying GammaTile to a secondary GB (grade 4 IDH-mutant astrocytoma) after previous surgery and irradiation to a grade 3 astrocytoma. A composite plan was successfully created between GammaTile and EBRT without hotspots or underdosing by creating a central gradient in the EBRT dose distribution that matched with GammaTile dose fall-off. In delivering a greater dose to resectable areas of disease than EBRT, GammaTile may result in improved PFS, and OS as has been seen with SRS for GB reirradiation. In the current case, we demonstrated that any potential benefit of dose escalation from GammaTile may still be achieved for tumors that are only partially resectable through pairing EBRT to residual disease significantly extending from the resection bed.
